# The urokinase plasminogen activation system in gastroesophageal cancer: A systematic review and meta-analysis

**DOI:** 10.18632/oncotarget.15485

**Published:** 2017-02-18

**Authors:** Daniel Brungs, Julia Chen, Morteza Aghmesheh, Kara L. Vine, Therese M. Becker, Martin G. Carolan, Marie Ranson

**Affiliations:** ^1^ Illawarra Health and Medical Research Institute, University of Wollongong, Wollongong, Australia; ^2^ School of Biological Sciences, University of Wollongong, Wollongong, Australia; ^3^ Illawarra Cancer Centre, Wollongong Hospital, Wollongong, Australia; ^4^ CONCERT-Translational Cancer Research Centre, New South Wales, Australia; ^5^ St George Cancer Centre, St George Hospital, Sydney, Australia; ^6^ Ingham Institute for Applied Medical Research, Liverpool Hospital, Australia; ^7^ School of Medicine, University of Western Sydney, Liverpool, Australia; ^8^ South Western Medical School, University of New South Wales, Liverpool, Australia

**Keywords:** stomach neoplasms, gastrointestinal neoplasms, urokinase-type plasminogen activator, urokinase plasminogen activator

## Abstract

**Background:**

The urokinase plasminogen activation (uPA) system is a crucial pathway for tumour invasion and establishment of metastasis. Although there is good evidence that uPA system expression is a clinically relevant biomarker in some solid tumours, its role in gastroesophageal cancer is uncertain.

**Results:**

We identified 22 studies encompassing 1966 patients which fulfilled the inclusion criteria. uPA, uPAR, or PAI-1 expression is significantly associated with high risk clinicopathological features. High uPA expression is associated with a shorter RFS (HR 1.90 95% 1.16–3.11, *p* = 0.01) and OS (HR 2.21 95% CI 1.74–2.80, *p* < 0.0001). High uPAR expression is associated with poorer OS (HR 2.21 95%CI 1.82–2.69, *p* < 0.0001). High PAI-1 expression is associated with shorter RFS (HR 1.96 96% CI 1.07–3.58, *p* = 0.03) and OS (HR 1.84 95%CI 1.28–2.64, *p* < 0.0001). There was no significant association between PAI-2 expression and OS (HR 0.97 95%CI 0.48–1.94, *p* < 0.92) although data was limited.

**Materials and Methods:**

We undertook a systematic review evaluating expression of uPA, urokinase plasminogen activator receptor (uPAR), plasminogen activator inhibitor-1 (PAI-1/SerpinE1) and plasminogen activator inhibitor-2 (PAI-2/SerpinB2) on primary oesophageal, gastro-oesophageal junction, and gastric adenocarcinomas. We performed a meta-analysis of clinicopathological associations, overall survival (OS) and recurrence free survival (RFS).

**Conclusions:**

We conclude that the uPA system is a clinically relevant biomarker in primary gastroesophageal cancer, with higher expression of uPA, uPAR and PAI-1 associated with higher risk disease and poorer prognosis. This also highlights the potential utility of the uPA system as a therapeutic target for improved treatment strategies.

## INTRODUCTION

Gastroesophageal cancer is a common and lethal malignancy, marked by modest response to systemic therapies [1]. A deeper understanding of molecular events characterising carcinogenesis, invasion, progression and metastasis is central for the development of novel therapies.

### The uPA system

**Figure 1:**
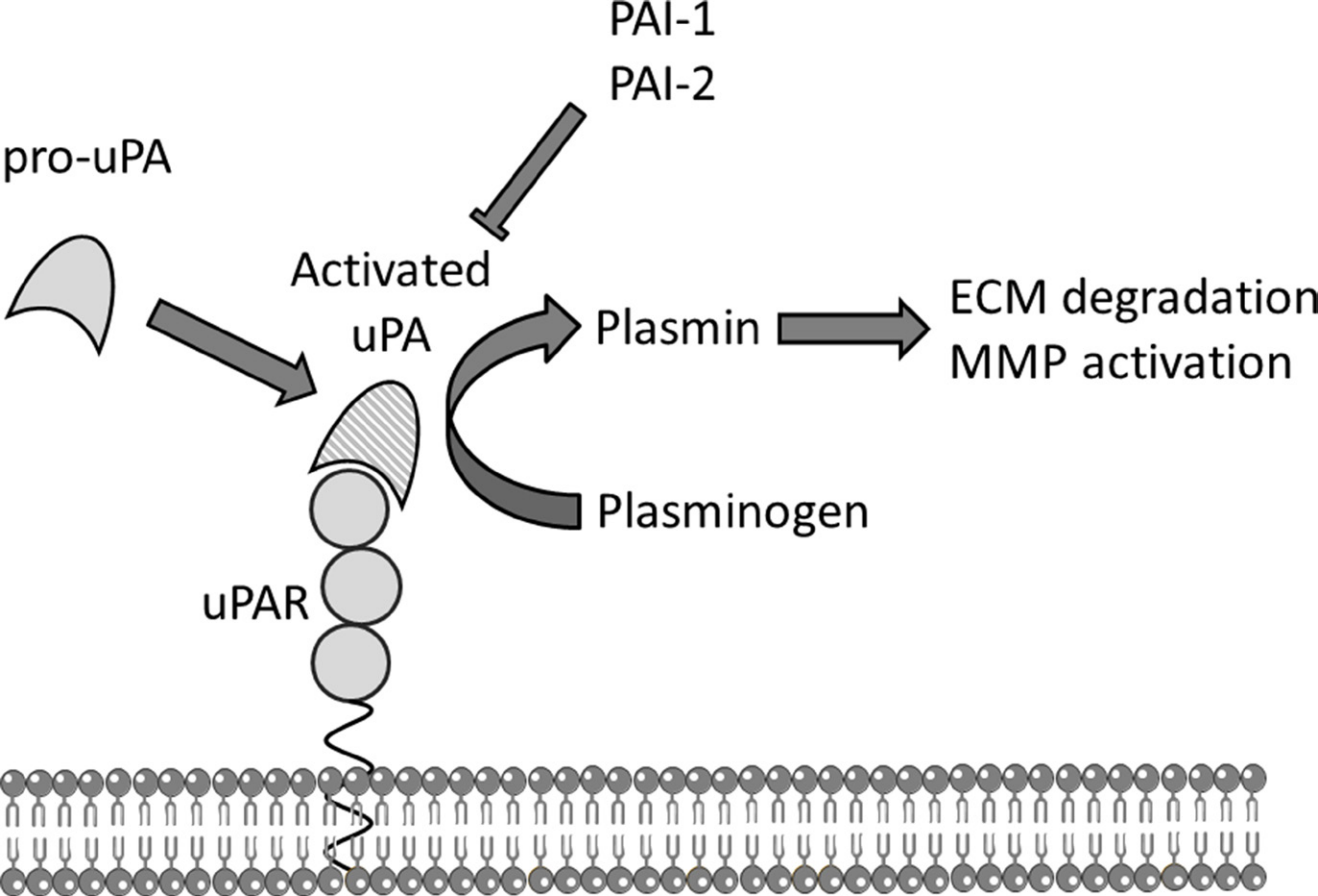
The uPA system Schematic representation of the urokinase plasminogen activation (uPA) system. The membrane bound urokinase receptor (uPAR) binds circulating inactive pro-uPA, facilitating the activation of pro-uPA to uPA which subsequently converts co-localised plasminogen to plasmin that can directly degrade components of the extracellular matrix (ECM) and activate pro-matrix metalloproteases (MMP) to further break down ECM. Plasminogen activator inhibitors 1 or 2 (PAI-1, PAI-2) are efficient endogenous inhibitors of uPA.

A key process in the development and progression of cancer, including establishment of metastatic disease, is the invasion of malignant cells into normal tissue. The plasminogen activation system, particularly the urokinase-type plasminogen activator (uPA) system, is critical for tumour-associated proteolysis to breakdown extracellular matrix (ECM) and basement membranes barriers [2]. The uPA system has a defined role in tissue degradation and extravascular fibrinolysis, and is responsible for most of the activated plasminogen associated with cancer invasion and metastasis [2, 3] (Figure 1).

The uPA protein is secreted as a zymogen and activated on high affinity binding to its specific cell surface receptor uPAR. Once activated, uPA catalyses the activation of co-localised plasminogen to plasmin, which in turn directly degrades components of the ECM, and promotes further degradation and tissue remodelling by activating pro-metalloproteinases and by releasing, thus activating, latent growth factors from the ECM [4].

The uPA receptor (uPAR) is anchored to the plasma membrane, localising the uPA system to the cell surface [5]. High expression of uPAR on the invasive front of tumours facilitates invasion and other roles in cellular migration and angiogenesis [6]. uPAR expression may be a suitable marker for the onset of invasion of both gastro-intestinal and breast cancer as it is expressed only on invasive carcinomas, not premalignant states such as Barrett's oesophagus [7].

Urokinase-type plasminogen activator is efficiently inhibited by two subtypes of serpin (serine proteinase inhibitor) family members, plasminogen activator inhibitor-1 (PAI-1/SerpinE1) and −2 (PAI-2 /SerpinB2). Both form a covalent complex with uPA/uPAR leading to internalisation of the entire complex [8]. Although believed to have a physiological role as an inhibitor of the uPA system, PAI-1 has a paradoxical protumourgenic role, increasing tumour invasion and angiogenesis, and correlated with poor prognosis [9]. The role of PAI-2 in cancer is less clear. Although both PAIs mediate uPA/uPAR endocytosis, the uPA-PAI-2 complex interacts with endocytosis receptors with different binding kinetics to those of uPA:PAI-1 and without stimulating intracellular signalling events over and above that of uPA binding to uPAR [10].

While the uPA system is expressed on both cancer cells and the supporting stroma, higher expression is seen on tumour cells, and is postulated that the tumour cell specific uPA/uPAR explains the aggressive biology exhibited by these cancers, and is more relevant for prognostic outcomes [11–14]. Expression of the uPA system has been shown to be an important prognostic marker in a variety of cancers including breast cancer [15], lung cancer [16], and colorectal cancer [17], with the combination of uPA and PAI-1 expression recommended to be incorporated into routine clinical care of node negative breast cancer [18].

In this study we aim to perform a comprehensive systematic review of expression of the uPA system encompassing uPA, uPAR, PAI-1, and PAI-2 in primary, resectable gastro-oesophageal cancer, and undertake meta-analyses of prognostic outcomes (recurrence free survival and overall survival), and association with relevant clinicopathological variables. To the best of our knowledge, this is the first meta-analysis to examine and compare the expression of these key components of uPA system in primary gastro-oesophageal cancer.

## RESULTS

### Included studies

**Figure 2:**
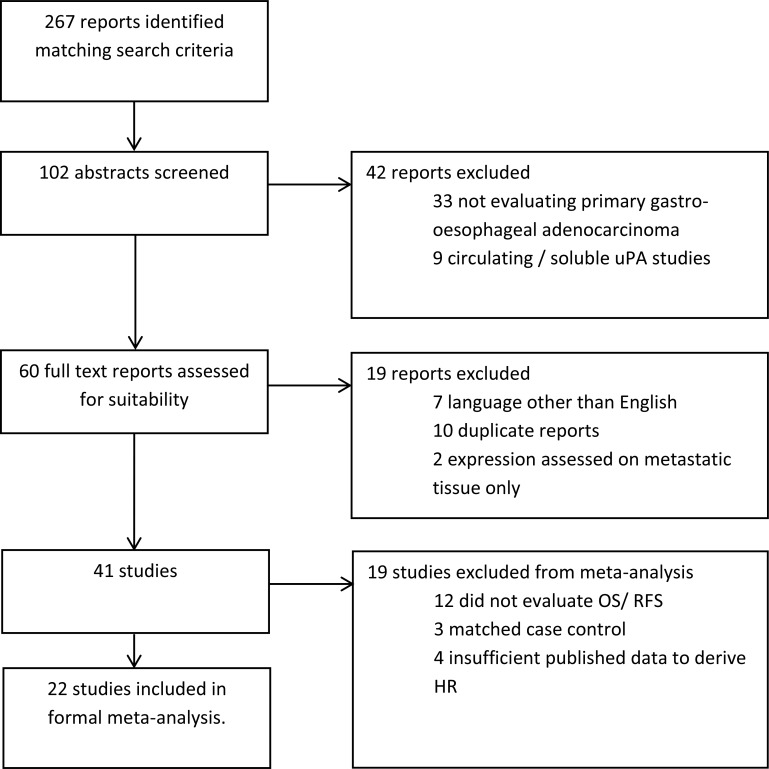
Study selection flow diagram HR –hazard ratio; OS–overall survival; RFS–recurrence free survival.

The trial flow is provided in Figure 2. We identified 267 reports matching criteria for inclusion in the study, of which 109 were selected for abstract review, and 60 subsequently for full text review. Forty one studies (including 2689 patients) fulfilled criteria for inclusion in the systematic review, with 22 studies (1966 patients) providing sufficient data for inclusion in the formal quantitative meta-analysis: 19 studies were excluded for the following reasons: 12 studies did not examine prognostic or clinicopathological associations, 3 reports were matched case control studies, and 4 studies reported insufficient published data to derive a HR.

The characteristics of the included studies are summarized in Supplementary Table 1. Eighteen studies evaluated uPA system expression in gastric cancer (1732 patients), one study included oesophageal, junctional and gastric cancers (39 patients), and two studies examined oesophageal cancer only (105 patients). Expression of the uPA system was assessed using immunohistochemistry (IHC, 12 studies, 1273 patients), enzyme-linked immunosorbent assay (ELISA, 5 studies, 344 patients), reverse transcription polymerase chain reaction (RT-PCR, 3 studies, 153 patients), or in-situ hybridisation (ISH, one study, 105 patients).

Hazard ratios directly extracted for 3 studies [7, 11, 22]. The multivariate HR was used when univariate value was not provided [22]. When only subgroup outcome data (tumour core or peripheral zone) were available, the results for peripheral “invasion” zone were used [7, 11]. Hazard ratios were estimated for the remaining studies using published data. 4 studies reported a “non-statistically significant OS” result for uPA system expression, but did not publish sufficient data for inclusion in meta-analysis [23–26].

### Bias risk

**Figure 3:**
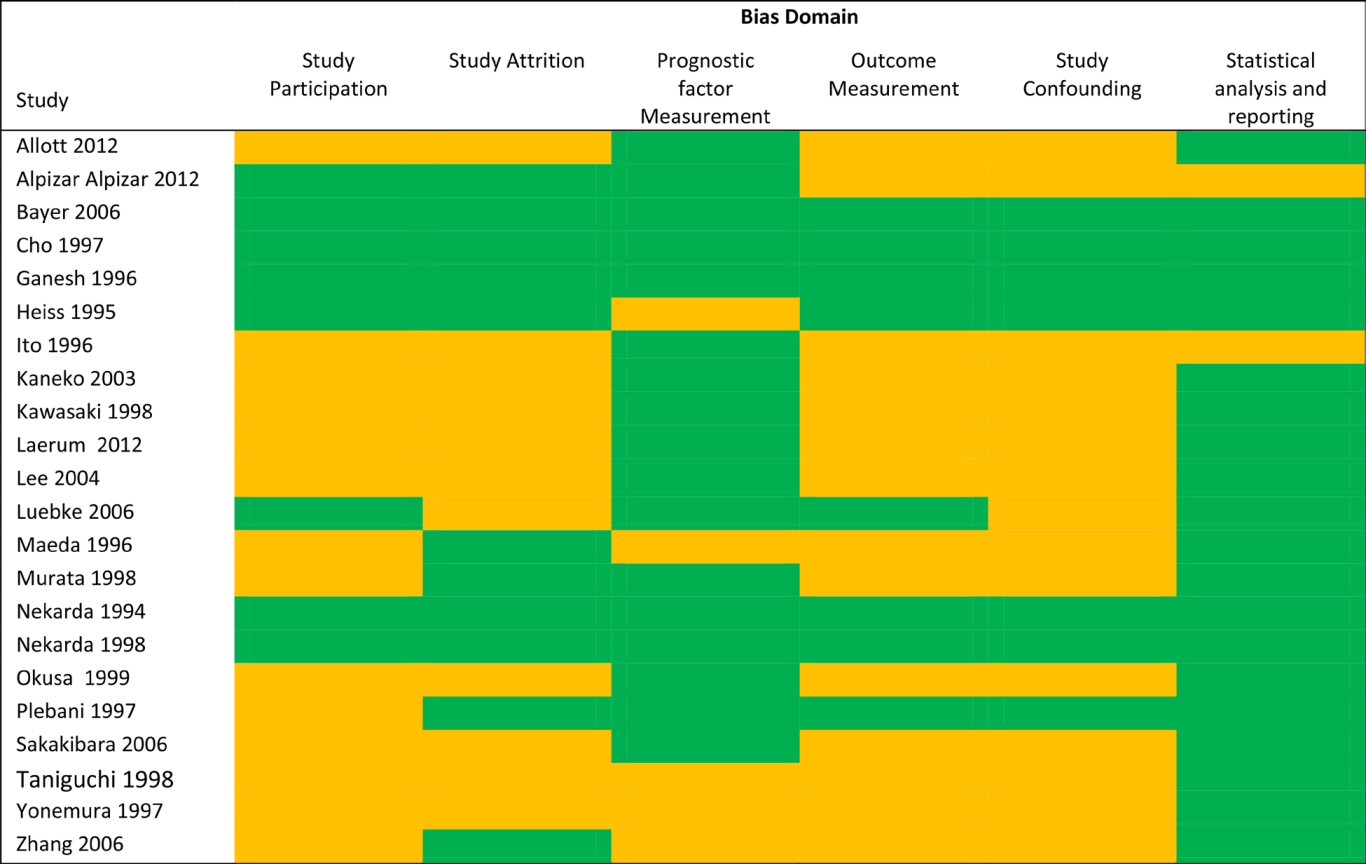
Risk of bias summary For each bias domain: green = “low risk” means that sufficient data was available to allow assessment of quality and fulfilled criteria for each domain, and accordingly is deemed low risk of bias. Orange = “unclear risk” means that insufficient data was presented to adequately assess the quality of the domain and accordingly the study has potentially high risk of bias. There were no studies deemed high risk of bias.

The risk of bias summary is summarized in Figure 3. Only 4 studies [22, 27–29] were deemed low risk in all bias domains. Fourteen studies did not clearly define the study population [7, 12, 13, 30–40] and 11 studies did not report completeness of followup [7, 12, 13, 30–33, 36, 38, 39, 41]. Most studies adequately reported method of measurement of the uPA system, although 5 studies did not report whether there was a second independent reviewer or blinding to clinical information [13, 35, 39, 40, 42]. The followup protocol was underreported in 14 studies [7, 11–13, 30–36, 38–40], although this is unlikely to bias the results for overall survival analyses. Most studies did not report details of the surgical, medical, or radiation treatments received by patients, and were Urokinase plasminogen activator (uPA).

### Urokinase plasminogen activator (uPA)

### uPA expression rates

Expression of uPA was evaluated in 13 studies (1254 patients). The mean expression of uPA was 52.8%, but had a large range (from 23% to 91%). There was no significant difference in mean expression for IHC (60.7%) and ELISA (45.6%) (*p* = 0.10).

### uPA and clinicopathological associations

uPA expression is significantly associated with poorer clinicopathological features in resected gastroesophageal cancer including: Advanced T stage (T3/4 vs T1/2) (OR 2.79 95% CI 1.80–4.32, *p* < 0.0001), nodal metastases (OR 2.30 95% CI 1.63–3.51, *p* < 0.0001), liver metastases (OR 6.77 95% CI 2.70–16.96, *p* < 0.0001), peritoneal metastases(OR 2.09 95% CI 1.29–3.36, *p* = 0.003), lymphatic invasion (OR 2.28 95% CI 1.31–3.97, *p* = 0.0003), and vascular invasion (OR = 2.43 95% CI 1.53–3.86, *p* = 0.0002) (5 studies, 522 patients, Supplementary Figure 1). There is no significant association with histology (poorly differentiated vs well differentiated).

### uPA expression and prognosis

uPA expression was significantly associated with a worse RFS (3 studies, 467 participants, HR 1.90 95% 1.16–3.11, *p* = 0.01) (see Supplementary Figure 2). There was no significant difference in RFS seen between studies using IHC (HR 1.77) or ELISA (HR 2.36) to assess uPA expression (test for subgroup differences Chi^2^ = 0.37, *p* = 0.54).

**Figure 4:**
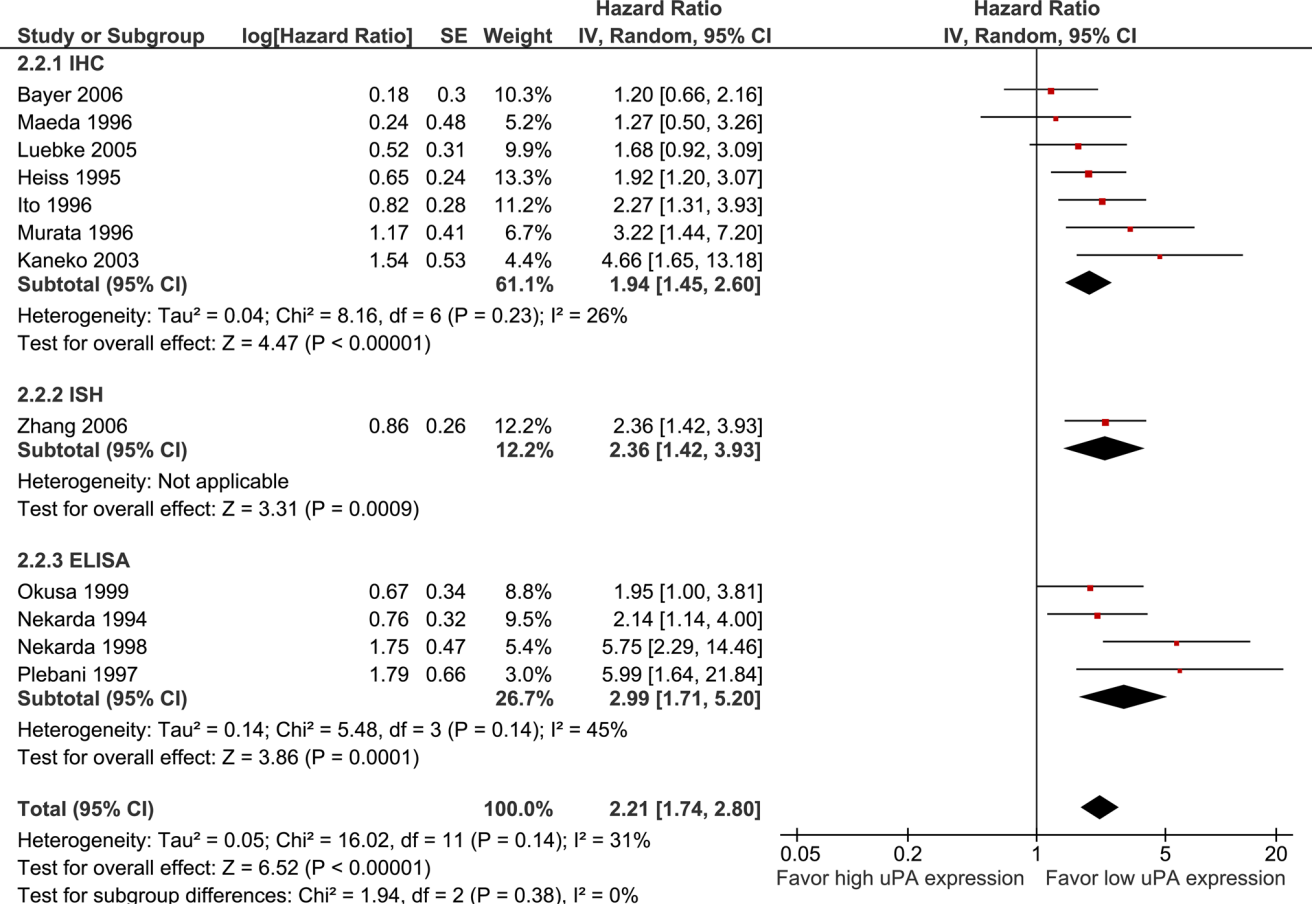
Pooled estimate of hazard ratio (HR) for uPA expression and overall survival (OS) Pooled estimate of hazard ratio (HR) for overall survival. The square on each bar represents the HR for an individual trial, and the bar shows the 95% confidence interval (CI). The diamond represents a pooled estimate with the centre of the diamond giving the HR estimate, and the extremes of the diamond representing the 95% CI. 24.

uPA expression is significantly associated with poorer OS (12 studies, 1094 participants, HR 2.21 95% CI 1.74–2.80, *p* < 0.0001) (see Figure 4). There was no significant difference in OS between studies which used IHC (HR 1.94) or ELISA (HR = 2.99) to assess uPA expression (*p* = 0.38). Sensitivity analysis showed similar results when analysis was restricted to gastric cancer only (HR 2.07, *p* < 0.00001).

### Urokinase plasminogen activator receptor (uPAR)

### uPAR expression rates

Twelve studies (1127 patients) evaluated uPAR expression, with mean uPAR expression of 56.8% (range 14–90%), with similar mean expressions seen in IHC (56.8%) and ELISA/RT-PCR (56.7%).

### uPAR expression and clinicopathological associations

uPAR expression on primary resected gastroesophageal cancer is significantly associated with poorer clinicopathological features including: advanced TMN stage (stage III/IV vs I/II, OR 3.41 91% CI 1.55–7.53, *p* = 0.002), advanced T stage (OR 2.33 95% CI 1.53 to 3.56, *p* < 0.0001), nodal metastases (OR 2.52 95% CI 1.70–3.72, *p* < 0.0001), liver metastases (OR 2.53 95% CI 1.25–5.13, *p* = 0.010), peritoneal metastases (OR 3.15 95% CI 1.87–5.28, *p* < 0.0001), lymphatic invasion (OR 2.82 95% CI 1.74–4.59, *p* < 0.0001) and vascular invasion (OR 3.85 95% CI 2.53–5.88, *p* < 0.0001) (six studies, 589 patients, Supplementary Figure 3). There is no significant association seen with histology (*p* = 0.6).

### uPAR expression and prognosis

Only one study provided data for uPAR expression and RFS [42], showing a shorter RFS with uPAR expression (203 patients, HR 2.69, *p* = 0.03).

**Figure 5:**
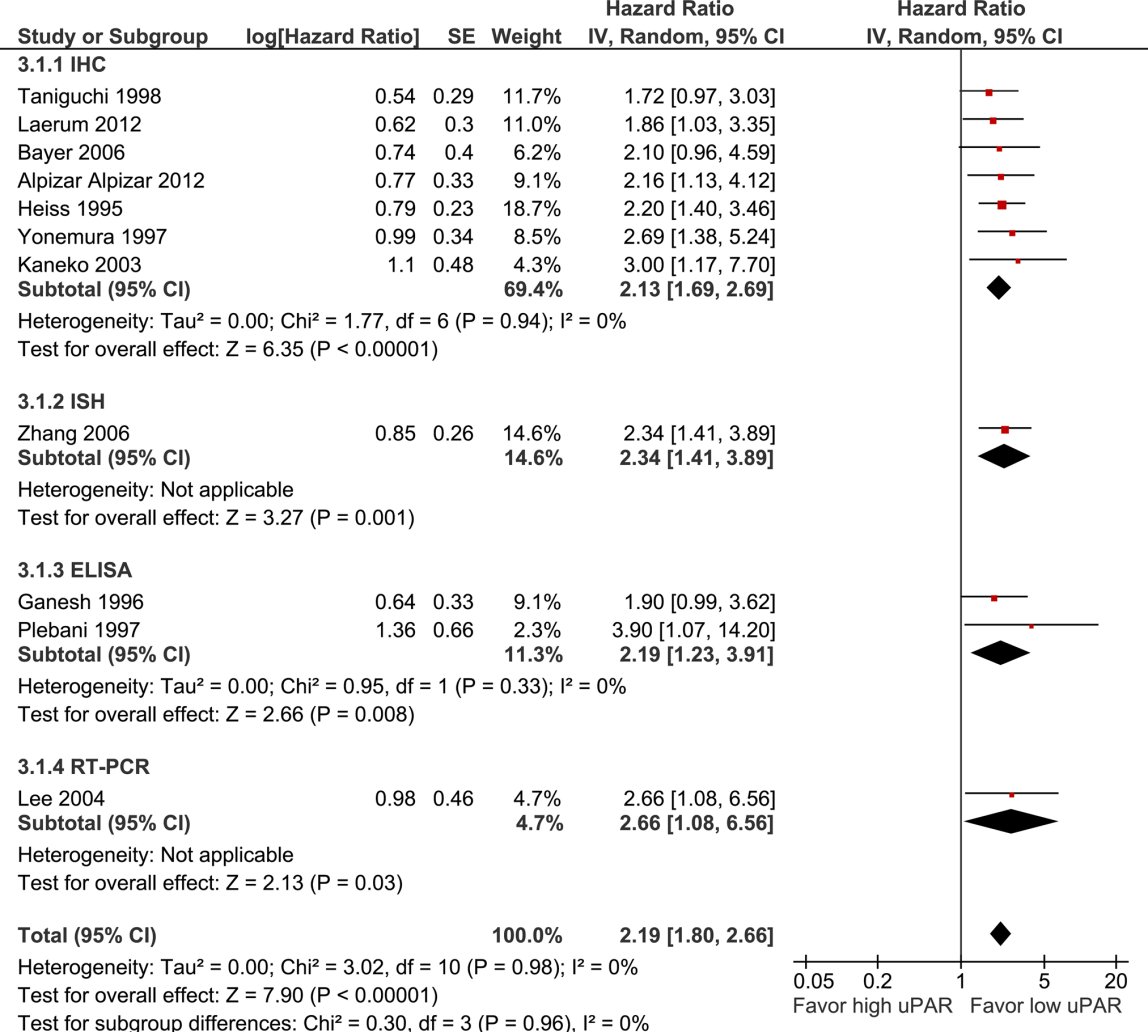
Pooled estimate of hazard ratio (HR) for uPAR expression and overall survival (OS)

uPAR expression is associated with poorer OS (11 studies, 1036 patients, HR 2.19 95% CI 1.80–2.66, *p* < 0.0001) (Figure 5). There was no significant difference in OS seen between studies which used IHC (HR 2.13), ISH (HR 2.34), ELISA (HR 2.19), or RT-PCR (2.66) to assess uPAR expression (*p* = 0.96).

### Plasminogen activator inhibitor-1 (PAI-1)

### PAI-1 expression rate

Twelve studies (1031 patients) examined PAI-1 expression. Mean PAI-1 expression was 53.3%, with no statically significant difference in expression between IHC (61.8%) and RT-PCR/ELISA (44.7%) (*p* = 0.1).

### PAI-1 expression and clinicopathological variables

PAI-1 expression on primary resected gastroesophageal cancer is significantly associated with poorer clinicopathological features including: advanced T stage (OR 2.59 95% CI 1.61 to 4.18, *p* < 0.0001), nodal metastases (OR 2.03 95% CI 1.27–3.22, *p* < 0.003), lymphatic invasion (OR 2.09 95% CI 1.31–3.34, *p* < 0.004) and vascular invasion (OR 1.90 95% CI 1.20–3.03, *p* < 0.007) (three studies, 317 patients, Supplementary Figure 4). There was no significant association of PAI-1 expression with presence of liver metastases (OR 0.52, *p* = 0.18), peritoneal metastases (OR 1.38, *p* = 0.31), or histology (OR 0.93, *p* = 0.74).

### PAI-1 expression and prognosis

PAI-1 expression is associated with shorter RFS (3 studies, 467 patients, HR 1.96 96% CI 1.07–3.58, *p* = 0.03) (Supplementary Figure 5). There was no significant difference in RFS between studies which used IHC or ELISA to detect PAI-1 expression (*p* = 0.86)

**Figure 6:**
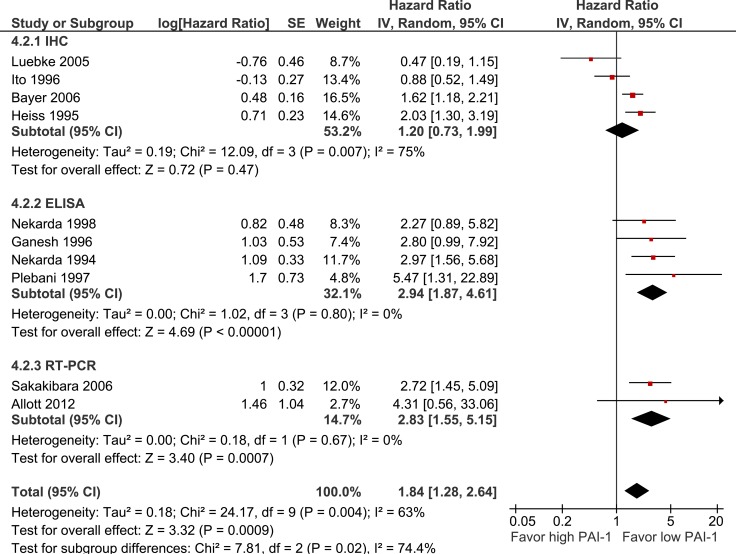
Pooled estimate of hazard ratio (HR) for PAI-1 expression and overall survival (OS)

PAI-1 expression is significantly associated with a shorter OS (10 studies, 839 participants, HR 1.84 95%CI 1.28–2.64, *p* < 0.0001, Figure 6). Pre-specified subgroup analysis showed a significant difference between studies which assessed PAI-1 expression using IHC (HR 1.20, *p* = 0.47) and ELISA (HR 2.94, *p* < 0.0001) or RT-PCR (HR 2.83, *p* < 0.0001) (*p* = 0.02).

### Plasminogen activator inhibitor-2 (PAI-2)

### PAI-2 expression rate

Two studies (145 participants) assessed PAI-2 expression (all using IHC) (refer to Supplementary Table 1). Mean expression was 57.5%.

### PAI-2 expression and clinicopathological variables

There were no studies with sufficient data analyzing PAI-2 expression and clinicopathological variables for inclusion in the meta-analysis.

### PAI-2 expression and prognosis

No studies published data on PAI-2 expression and RFS. There was no significant association of PAI-2 expression and OS (2 studies, 145 participants, HR 0.97 95%CI 0.48–1.94, *p* < 0.92, Supplementary Figure 6).

### Publication bias

Examination of the funnel plots for the OS analysis for uPA, uPAR and PAI-1 showed asymmetrical plots for all analyses, suggesting absence of smaller negative trials (example plot for uPA provided in Supplementary Figure 7).

## DISCUSSION

This meta-analysis confirms the clinical utility of the uPA system as a biomarker in resected gastro-oesophageal adenocarcinoma.

There is good evidence that high expression of uPA, uPAR, and PAI-1 is associated with most high risk clinicopathological features, including advanced T stage, presence of nodal and distant metastases, and lymphovascular invasion, in primary gastro-oesophageal adenocarcinoma. This supports the central role of the uPA system in tumour invasion and metastasis. In contrast, there was no significant association of expression found with poorly differentiated histology, consistent with previously published work which shows that epithelial cell uPA system expression is higher in malignant than benign tissue, but decreases as tumour becomes more poorly differentiated, with a corresponding increase in stromal expression [43].

We also demonstrated that uPA, uPAR, and PAI-1 expression is associated with poorer prognosis in resected gastro-oesophageal cancer, with both a shorter RFS and OS in tumours which expressed these markers. However this result should be interpreted with caution due to the following important limitations in our study.

Firstly, only four of the included studies were deemed low risk for all bias domains as assessed by the QUIPS tool. In particular, most studies did not report the treatments patients received which is an important potential source of confounding for RFS and OS analyses. Additionally, tumours with higher risk clinicopathologic features could reasonably be expected to be more likely to have received neoadjuvant treatment prior to surgery, which may in turn have impacted on the expression of the uPA system. Despite this, it should be noted that similar results were seen in studies deemed low and high risk of study confounding, and heterogeneity was low in both the uPA and uPAR OS meta-analyses (I^2^ = 31% and 0% respectively, see Figures 4 and 5).

Secondly, there is evidence of underreporting of non-significant results. This is demonstrated by both the funnel plot, as well the selective reporting of only statistically positive findings from included studies. This important bias will cause an overestimation of the effect of expression.

Thirdly, as demonstrated above, tumours that expressed uPA, uPAR and PAI-1 had higher risk features, and would be expected to recur or progress sooner than tumours that did not. The apparent difference in prognostic outcomes may be due to unequal baseline characteristics of the included participants.

We did not show a significant difference in the prognostic outcomes between studies which used a tumour cell specific technique (e.g. IHC) compared to whole tissue lysates (e.g. RT-PCR, ELISA) for uPA and uPAR. This is consistent with other studies which have shown correlation between IHC score and median ELISA value, and supports the cancer cells as a major source of uPA and uPAR expression in the tumour tissue [44].

In contrast, there was a significant different in the expression methodology subgroups in the analysis for PAI-1 and OS (*p* = 0.02), with a non-significant outcome seen in studies using IHC (HR 1.20, *p* = 0.47), compared to significant results with ELISA (HR 2.94, *p* < 0.0001) and RT-PCR (HR 2.83, *p* = 0.0007). This highlights the importance of the stromal production of PAI-1 within the tumour microenvironment [9], as only methods that took into account both stromal and tumour PAI-1 showed statistically significant prognostic outcomes. It has been postulated that in contrast to uPAR, fibroblasts and endothelial cells provide the major source of PAI-1 within the tumour tissue [45]. It is possible that the PAI-1 detected on the tumour cells by IHC may be explained by internalization and accumulation of stromal produced uPA-PAI-1 complexes mediated by tumour uPAR [46]. No IHC studies examined the association between stromal PAI-1 expression and prognostic outcomes in gastro-oesophageal cancer.

All IHC study results used in the meta-analysis were restricted to tumour cell expression only. Similar to other cancers, uPA system expression was highest at the invasive front of the tumour [7, 11, 12, 31]. Only four studies reported stromal expression of the uPA system [7, 11, 12, 42]. Results were conflicting, with only one study showing a significant association of OS with macrophage uPAR expression on the invading zone at the periphery of the tumour [7]. In colorectal cancer, high uPAR expression on macrophages in the tumour core, rather than the periphery, is an independent predictor of poor prognosis [47]. These studies suggest an important supporting role of the tumour associated macrophages within the tumour microenvironment. The contrasting pattern of high uPAR expression (core versus peripheral) may be due to differing phenotypes of the subpopulations of tumour preventing (M1 macrophages) and tumour promoting (M2 macrophages) macrophages within the heterogeneous tumour bulk [48]. Further work is required to elucidate the biology of the stroma in gastrointestinal cancers.

We were unable to show any significant associations with PAI-2 expression with either clinicopathological features or prognostic outcomes, as available data was much more limited. Similarly only 3 studies examined oesophageal cancer, which limits applicability of our results to this subgroup. Sensitivity analysis did not show a different result when oesophageal cancer was excluded from analysis.

In conclusion, expression of the uPA system is a clinically relevant biomarker in gastroesophageal cancer. There is good evidence to support the association of uPA, uPAR, and PAI-1 expression and high risk clinicopathological features. While we found a statistically significant association between uPAR, uPAR and PAI-1 expression and poorer prognosis, our results are tempered by methodical limitations discussed above. Our findings also highlight the potential utility of the uPA system as a therapeutic target for improved treatment strategies.

## MATERIALS AND METHODS

Methods are reported according to Preferred Reporting for Systematic Reviews and Meta-Analyses (PRISMA) guidelines [19].

### Study eligibility/selection criteria

We included all studies which examined the following components of the urokinase plasminogen activation system uPA, uPAR, PAI-1 or PAI-2, in resected primary esophageal, gastroesophageal junction, or gastric adenocarcinomas. Other tumour pathologies were excluded. A ll methods of assessing expression, including reverse transcription polymerase chain reaction (RT-PCR), enzyme-linked immunosorbent assay (ELISA), in-situ hybridization (ISH), and immunohistochemistry (IHC) were included. For inclusion in the meta-analysis, studies were required to report the association of the following outcomes with uPA system expression: overall survival (OS), recurrence-free survival (RFS), or clinicopathological variables.

Two authors (DB, JC) independently performed the search and screened the studies. The primary outcome was OS; secondary outcomes were RFS, and correlation of clinicopathological variables with uPA system expression.

### Study search strategy

We searched the following databases in February 2015 for all trials fulfilling the above criteria: Medline (1950–present); EMBASE (1966–present); Cochrane Central Register of Controlled Trials, and Cochrane Database of Systematic Reviews; PubMed.

To maximize sensitivity the following search terms were used: Stomach Neoplasms (MESH) OR Esophageal neoplasms (MESH) OR Gastrointestinal neoplasms (MESH) OR Gastric cancer.mp OR Gastric carcinoma. mp OR esophageal cancer.mp OR oesophageal cancer. mp OR gastroesophageal cancer.mp AND Receptors, urokinase plasminogen activator (MESH) OR Urokinase-type plasminogen activator (MESH) OR Plasminogen activator inhibitor 1 (MESH) OR Plasminogen activator inhibitor.mp OR PAI-1.mp OR PAI-2.mp OR Urokinase* plasminogen.mp OR uPA*.mp. Reference lists of included studies and review articles were hand searched. The search was restricted to studies published in English.

### Data collection

Study data was independently collected by two authors (DB, JC) using standardized electronic data collection forms. The following was collected for each study: patient number, primary tumour location (gastric/oesophageal/COJ), cancer stage, treatment received by patient; uPA components assessed (uPA, uPAR, PAI- 1, PAI-2) and method, patient followup; outcomes (OS or RFS), clinicopathological correlations (including TMN stage, tumour grade, lymphatic invasion, vascular invasion). For studies which used IHC, expression analysis was restricted to tumour cells only (stromal expression was not included in the meta-analysis).

### Assessment of bias within studies

All studies included in the meta-analyses were assessed for bias using the Quality In Prognosis Studies (QUIPS) tool which assesses for potential sources of bias in six domains namely: study participation; study attrition and loss to followup; prognostic factor measurement; outcome measurement; study confounding; and statistical analysis and reporting [20].

### Statistical analysis

We extracted the hazard ratio (HR) and their 95% confidence intervals (CI) for time-to-event outcomes including RFS and OS. If both univariate and multivariate HR were published the univariate results were preferentially used. Where no HR was provided in published data, it was estimated from available results or Kaplan-Meier survival curves using previously described methods [21].

HRs were synthesized using the generic inverse variance method and a random effect model using RevMan5.1 analysis software. Statistical heterogeneity was assessed using the I^2^ statistic. We performed pre-specified subgroup analysis for overall survival for: primary location (gastric or oesophageal), cancer cell specific expression (using IHC) compared to whole cell lysis (using RT-PCR/ELISA).

Clinicopathological associations were summarized using odds ratios (OR) derived from published results. This analysis was limited to studies using IHC, as other methods presented expression results as means, rather than percentage of patients expressing. Expression rates were described with mean and range, and compared using the student's *t*-test.

## SUPPLEMENTARY MATERIALS FIGURES AND TABLE


