# Supplementary material for: The urokinase plasminogen activation system in gastroesophageal cancer: A systematic review and meta-analysis
Source: Oncotarget. 2017 Feb 18;8(14):23099–109. doi: 10.18632/oncotarget.15485 (PMC5410288; doi:10.18632/oncotarget.15485)
Supplement: Supplementary file 1 [file oncotarget-08-23099-s001.pdf]

The urokinase plasminogen activation system in gastroesophageal cancer: A systematic review and meta-analysis

Supplementary Materials

Study or Subgroup	uPA positive Events	uPA positive Total	uPA negative Events	uPA negative Total	Weight	Odds Ratio M-H, Fixed, 95% CI
**1.1.1 Advanced T Stage (T3/4 vs T1/2)**						
Okusa 1999	8	16	11	16	23.0%	0.45 [0.11, 1.92]
Ito 1996	62	82	24	43	32.1%	2.45 [1.12, 5.38]
Zhang 2006	44	61	17	44	23.0%	4.11 [1.80, 9.39]
Kaneko 2003	35	49	19	52	22.0%	4.34 [1.88, 10.04]
**Subtotal (95% CI)**		**208**		**155**	**100.0%**	**2.79 [1.80, 4.32]**
Total events	149		71			
Heterogeneity: Chi² = 8.11, df = 3 (P = 0.04); I² = 63%						
Test for overall effect: Z = 4.60 (P < 0.00001)						
**1.1.2 Nodal Metastases**						
Okusa 1999	13	16	12	16	6.7%	1.44 [0.27, 7.83]
Maeda 1996	47	73	23	47	29.9%	1.89 [0.89, 3.98]
Kaneko 2003	22	49	15	52	24.0%	2.01 [0.88, 4.58]
Zhang 2006	39	53	23	44	19.9%	2.54 [1.09, 5.95]
Ito 1996	55	82	15	43	19.4%	3.80 [1.75, 8.28]
**Subtotal (95% CI)**		**273**		**202**	**100.0%**	**2.39 [1.63, 3.51]**
Total events	176		88			
Heterogeneity: Chi² = 2.29, df = 4 (P = 0.68); I² = 0%						
Test for overall effect: Z = 4.43 (P < 0.00001)						
**1.1.3 Liver Metastases**						
Okusa 1999	1	16	1	16	19.3%	1.00 [0.06, 17.51]
Ito 1996	10	82	1	43	23.7%	5.83 [0.72, 47.19]
Zhang 2006	15	39	3	42	36.7%	8.13 [2.13, 31.02]
Maeda 1996	14	73	1	47	20.3%	10.92 [1.38, 86.08]
**Subtotal (95% CI)**		**210**		**148**	**100.0%**	**6.77 [2.70, 16.96]**
Total events	40		6			
Heterogeneity: Chi² = 2.01, df = 3 (P = 0.57); I² = 0%						
Test for overall effect: Z = 4.08 (P < 0.0001)						
**1.1.4 Peritoneal Metastases**						
Okusa 1999	4	16	6	16	18.4%	0.56 [0.12, 2.54]
Kaneko 2003	5	49	9	57	30.6%	0.61 [0.19, 1.95]
Maeda 1996	13	73	6	47	24.5%	1.48 [0.52, 4.21]
Ito 1996	26	82	6	43	22.0%	2.86 [1.07, 7.63]
Zhang 2006	22	46	2	41	4.5%	17.88 [3.85, 82.90]
**Subtotal (95% CI)**		**266**		**204**	**100.0%**	**2.09 [1.29, 3.36]**
Total events	70		29			
Heterogeneity: Chi² = 15.57, df = 4 (P = 0.004); I² = 74%						
Test for overall effect: Z = 3.02 (P = 0.003)						
**1.1.5 Undifferentiated Histology**						
Kaneko 2003	17	49	35	52	40.6%	0.26 [0.11, 0.59]
Okusa 1999	9	16	13	16	10.4%	0.30 [0.06, 1.47]
Maeda 1996	39	73	31	47	32.1%	0.59 [0.28, 1.26]
Ito 1996	50	82	18	43	16.9%	2.17 [1.02, 4.60]
**Subtotal (95% CI)**		**220**		**158**	**100.0%**	**0.69 [0.46, 1.04]**
Total events	115		97			
Heterogeneity: Chi² = 15.62, df = 3 (P = 0.001); I² = 81%						
Test for overall effect: Z = 1.75 (P = 0.08)						
**1.1.6 Lymphatic Invasion**						
Ito 1996	57	82	25	43	61.2%	1.64 [0.76, 3.53]
Kaneko 2003	33	49	20	52	38.8%	3.30 [1.46, 7.48]
**Subtotal (95% CI)**		**131**		**95**	**100.0%**	**2.28 [1.31, 3.97]**
Total events	90		45			
Heterogeneity: Chi² = 1.49, df = 1 (P = 0.22); I² = 33%						
Test for overall effect: Z = 2.93 (P = 0.003)						
**1.1.7 Vascular Invasion**						
Ito 1996	38	82	17	43	52.0%	1.32 [0.62, 2.80]
Zhang 2006	48	61	28	44	30.1%	2.11 [0.89, 5.02]
Kaneko 2003	33	49	13	52	17.9%	6.19 [2.60, 14.71]
**Subtotal (95% CI)**		**192**		**139**	**100.0%**	**2.43 [1.53, 3.86]**
Total events	119		58			
Heterogeneity: Chi² = 7.11, df = 2 (P = 0.03); I² = 72%						
Test for overall effect: Z = 3.75 (P = 0.0002)						

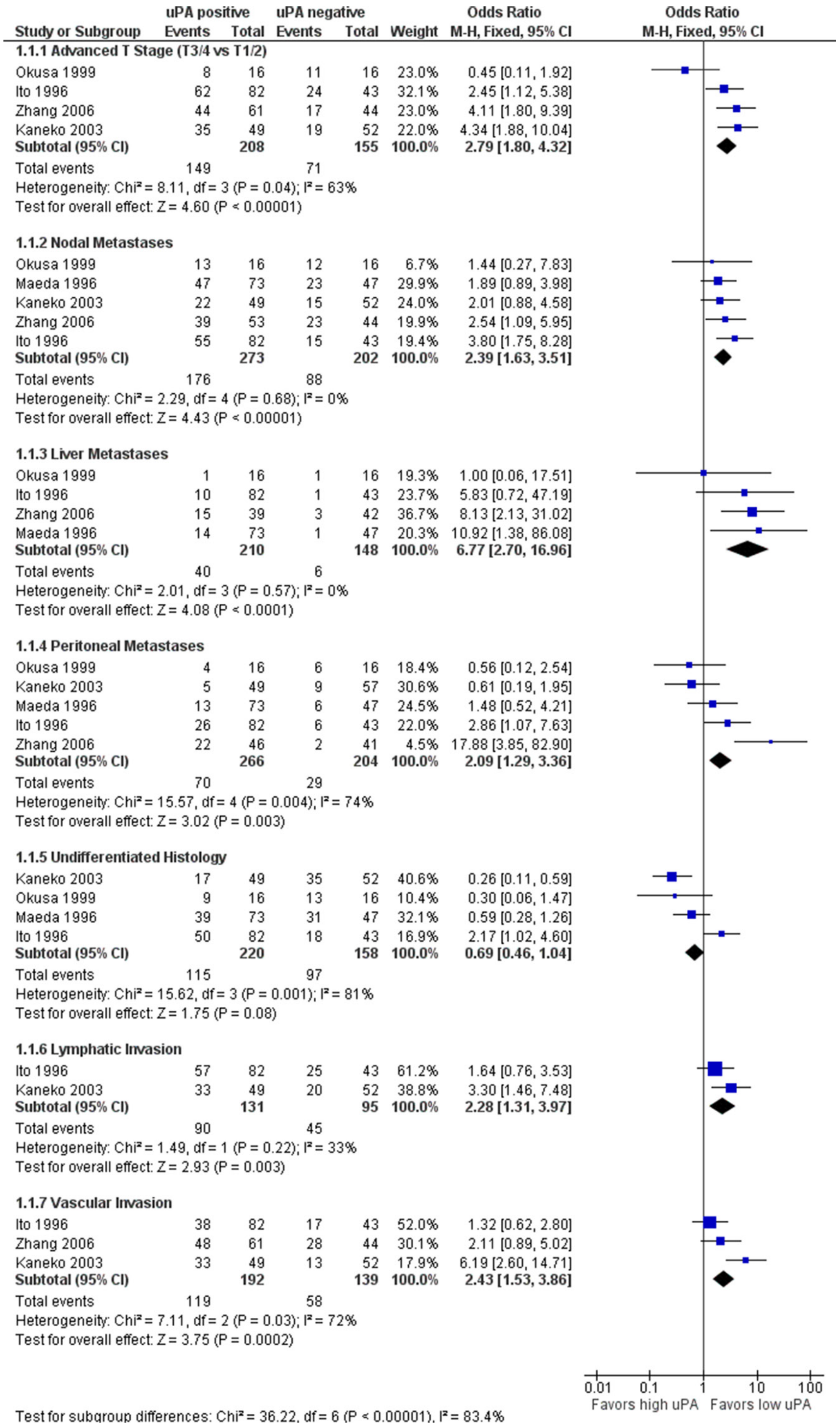

Test for subgroup differences: Chi² = 36.22, df = 6 (P < 0.00001), I² = 83.4%

Supplementary Figure 1: Pooled estimate of odds ratio (OR) for uPA expression and clinicopathological factors.

Study or Subgroup	log[Hazard Ratio]	SE	Weight	Hazard Ratio IV, Random, 95% CI
**2.1.1 IHC**				
Bayer 2006	0.26	0.19	42.3%	1.30 [0.89, 1.88]
Heiss 1995	0.99	0.34	27.6%	2.69 [1.38, 5.24]
**Subtotal (95% CI)**			**69.9%**	**1.77 [0.87, 3.59]**
Heterogeneity: Tau² = 0.19; Chi² = 3.51, df = 1 (P = 0.06); I² = 72%				
Test for overall effect: Z = 1.58 (P = 0.11)				
**2.1.2 ELISA**				
Cho 1997	0.86	0.31	30.1%	2.36 [1.29, 4.34]
**Subtotal (95% CI)**			**30.1%**	**2.36 [1.29, 4.34]**
Heterogeneity: Not applicable				
Test for overall effect: Z = 2.77 (P = 0.006)				
**Total (95% CI)**			**100.0%**	**1.90 [1.16, 3.11]**
Heterogeneity: Tau² = 0.11; Chi² = 4.98, df = 2 (P = 0.08); I² = 60%				
Test for overall effect: Z = 2.56 (P = 0.01)				
Test for subgroup differences: Chi² = 0.37, df = 1 (P = 0.54), I² = 0%				

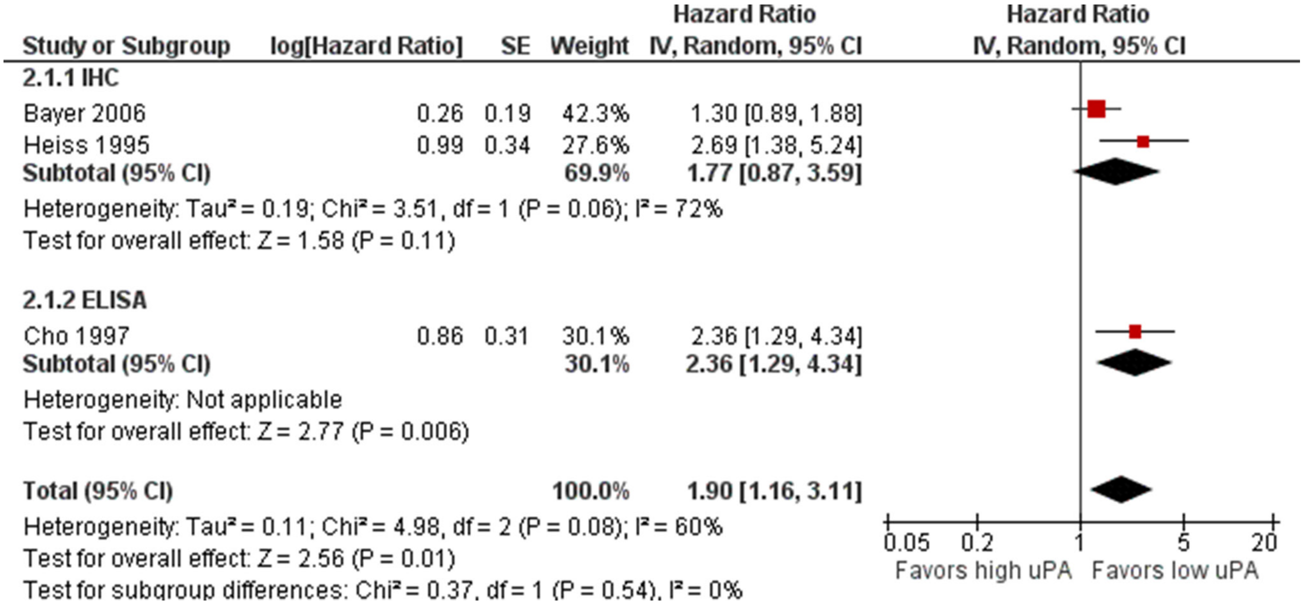

Supplementary Figure 2: Pooled estimate of hazard ratio (HR) for uPA expression and recurrence free survival (RFS).

Study or Subgroup	uPAR positive Events	uPAR positive Total	uPAR negative Events	uPAR negative Total	Weight	Odds Ratio M-H, Fixed, 95% CI
**1.2.1 Advanced TNM Stage (III/IV vs I/II)**						
Yonemura 1997	12	21	50	134	89.4%	2.24 [0.88, 5.69]
Lee 2004	20	24	3	11	10.6%	13.33 [2.42, 73.48]
**Subtotal (95% CI)**		**45**		**145**	**100.0%**	**3.41 [1.55, 7.53]**
Total events	32		53			
Heterogeneity: Chi² = 3.23, df = 1 (P = 0.07); I² = 69%						
Test for overall effect: Z = 3.04 (P = 0.002)						
**1.2.2 Advanced T stage (T3/4 vs T1/2)**						
Zhang 2006	16	40	15	35	34.7%	0.89 [0.35, 2.23]
Yonemura 1997	8	21	37	134	22.4%	1.61 [0.62, 4.21]
Taniguchi 1998	19	41	12	61	18.7%	3.53 [1.46, 8.51]
Lee 2004	18	24	5	11	6.2%	3.60 [0.80, 16.20]
Kaneko 2003	42	63	12	38	18.0%	4.33 [1.83, 10.26]
**Subtotal (95% CI)**		**189**		**279**	**100.0%**	**2.33 [1.53, 3.56]**
Total events	103		81			
Heterogeneity: Chi² = 7.93, df = 4 (P = 0.09); I² = 50%						
Test for overall effect: Z = 3.94 (P < 0.0001)						
**1.2.3 Nodal Metastases**						
Taniguchi 1998	25	41	30	61	29.3%	1.61 [0.72, 3.61]
Yonemura 1997	15	21	72	134	17.4%	2.15 [0.79, 5.89]
Kawaski 1998	17	30	20	61	17.8%	2.68 [1.09, 6.58]
Kaneko 2003	20	53	7	38	15.8%	2.68 [1.00, 7.23]
Zhang 2006	62	80	18	35	17.6%	3.25 [1.40, 7.58]
Lee 2004	22	24	6	11	2.1%	9.17 [1.41, 59.59]
**Subtotal (95% CI)**		**249**		**340**	**100.0%**	**2.52 [1.70, 3.72]**
Total events	161		153			
Heterogeneity: Chi² = 3.49, df = 5 (P = 0.63); I² = 0%						
Test for overall effect: Z = 4.62 (P < 0.00001)						
**1.2.4 Liver Metastases**						
Yonemura 1997	2	21	10	134	25.8%	1.31 [0.27, 6.42]
Kawaski 1998	5	30	4	61	23.2%	2.85 [0.71, 11.51]
Zhang 2006	14	48	4	33	35.4%	2.99 [0.88, 10.08]
Taniguchi 1998	4	41	2	59	15.6%	3.08 [0.54, 17.68]
**Subtotal (95% CI)**		**140**		**287**	**100.0%**	**2.53 [1.25, 5.13]**
Total events	25		20			
Heterogeneity: Chi² = 0.81, df = 3 (P = 0.85); I² = 0%						
Test for overall effect: Z = 2.58 (P = 0.010)						
**1.2.5 Peritoneal Metastases**						
Kawaski 1998	5	30	6	61	20.4%	1.83 [0.51, 6.58]
Kaneko 2003	7	63	2	38	13.7%	2.25 [0.44, 11.44]
Yonemura 1997	7	21	22	134	24.6%	2.55 [0.92, 7.03]
Taniguchi 1998	15	41	10	61	31.6%	2.94 [1.16, 7.45]
Zhang 2006	22	56	2	31	9.7%	9.38 [2.03, 43.33]
**Subtotal (95% CI)**		**211**		**325**	**100.0%**	**3.15 [1.87, 5.28]**
Total events	56		42			
Heterogeneity: Chi² = 3.00, df = 4 (P = 0.56); I² = 0%						
Test for overall effect: Z = 4.33 (P < 0.0001)						
**1.2.6 Undifferentiated Histology**						
Kaneko 2003	26	63	26	38	44.0%	0.32 [0.14, 0.76]
Kawaski 1998	17	30	41	61	27.1%	0.64 [0.26, 1.57]
Lee 2004	3	24	1	11	2.8%	1.43 [0.13, 15.52]
Taniguchi 1998	28	41	30	61	17.7%	2.23 [0.97, 5.09]
Yonemura 1997	16	21	57	134	8.5%	4.32 [1.50, 12.49]
**Subtotal (95% CI)**		**179**		**305**	**100.0%**	**1.12 [0.74, 1.68]**
Total events	90		155			
Heterogeneity: Chi² = 18.61, df = 4 (P = 0.0009); I² = 79%						
Test for overall effect: Z = 0.52 (P = 0.60)						
**1.2.7 Lymphatic Invasion**						
Lee 2004	6	24	4	11	20.4%	0.58 [0.13, 2.71]
Kaneko 2003	38	63	15	38	36.8%	2.33 [1.02, 5.31]
Taniguchi 1998	31	41	26	61	25.3%	4.17 [1.74, 10.01]
Kawaski 1998	25	30	32	61	17.4%	4.53 [1.53, 13.39]
**Subtotal (95% CI)**		**158**		**171**	**100.0%**	**2.82 [1.74, 4.59]**
Total events	100		77			
Heterogeneity: Chi² = 5.75, df = 3 (P = 0.12); I² = 48%						
Test for overall effect: Z = 4.18 (P < 0.0001)						
**1.2.8 Vascular Invasion**						
Lee 2004	18	24	7	11	11.1%	1.71 [0.37, 7.97]
Zhang 2006	55	70	21	35	27.6%	2.44 [1.01, 5.92]
Taniguchi 1998	22	41	19	61	32.6%	2.56 [1.13, 5.80]
Kawaski 1998	23	30	25	61	17.7%	4.73 [1.76, 12.71]
Kaneko 2003	48	63	8	38	10.9%	12.00 [4.54, 31.71]
**Subtotal (95% CI)**		**228**		**206**	**100.0%**	**3.85 [2.53, 5.88]**
Total events	166		80			
Heterogeneity: Chi² = 8.46, df = 4 (P = 0.08); I² = 53%						
Test for overall effect: Z = 6.26 (P < 0.00001)						

Odds Ratio
M-H, Fixed, 95% CI

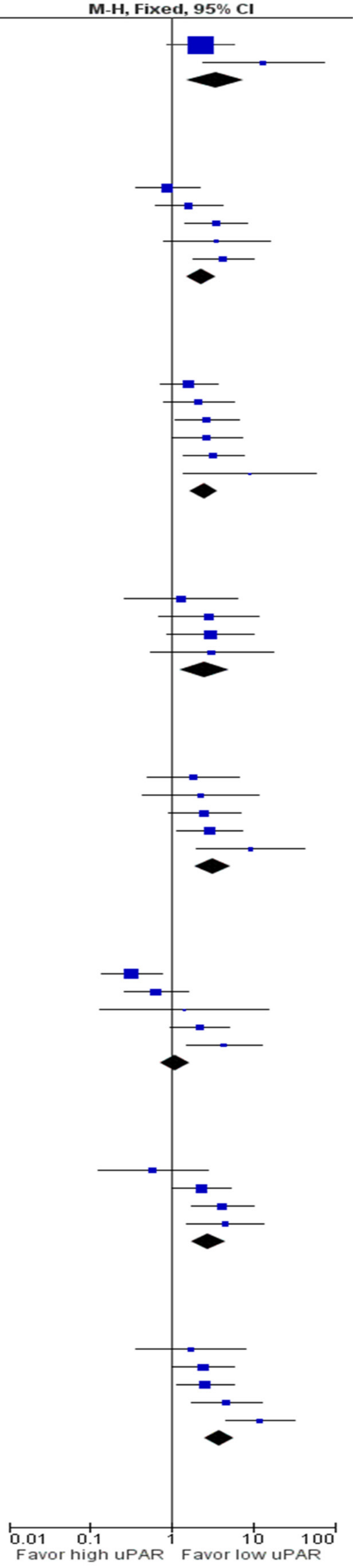

Test for subgroup differences: Chi² = 20.50, df = 7 (P = 0.005), I² = 65.9%

Supplementary Figure 3: Pooled estimate of odds ratio (OR) for uPAR expression and clinicopathological factors.

Study or Subgroup	PAI-1 positive Events	PAI-1 positive Total	PAI-1 negative Events	PAI-1 negative Total	Weight	Odds Ratio M-H, Fixed, 95% CI
**1.3.1 Advanced T stage (T3/T4 vs T1/T2)**						
Ito 1996	44	62	42	63	57.1%	1.22 [0.57, 2.61]
Kaneko 2003	43	63	11	38	20.5%	5.28 [2.19, 12.71]
Kawaski 1998	27	35	27	56	22.4%	3.63 [1.41, 9.35]
**Subtotal (95% CI)**		**160**		**157**	**100.0%**	**2.59 [1.61, 4.18]**
Total events	114		80			
Heterogeneity: Chi² = 6.77, df = 2 (P = 0.03); I² = 70%						
Test for overall effect: Z = 3.90 (P < 0.0001)						
**1.3.2 Nodal Metastases**						
Ito 1996	35	62	35	63	59.8%	1.04 [0.51, 2.10]
Kaneko 2003	30	63	7	38	18.1%	4.03 [1.55, 10.49]
Kawaski 1998	20	35	17	56	22.2%	3.06 [1.27, 7.37]
**Subtotal (95% CI)**		**160**		**157**	**100.0%**	**2.03 [1.27, 3.22]**
Total events	85		59			
Heterogeneity: Chi² = 6.27, df = 2 (P = 0.04); I² = 68%						
Test for overall effect: Z = 2.98 (P = 0.003)						
**1.3.3 Liver Metastases**						
Ito 1996	2	62	9	63	71.7%	0.20 [0.04, 0.97]
Kawaski 1998	4	35	5	56	28.3%	1.32 [0.33, 5.28]
**Subtotal (95% CI)**		**97**		**119**	**100.0%**	**0.52 [0.19, 1.37]**
Total events	6		14			
Heterogeneity: Chi² = 3.14, df = 1 (P = 0.08); I² = 68%						
Test for overall effect: Z = 1.33 (P = 0.18)						
**1.3.4 Peritoneal Metastases**						
Ito 1996	16	62	16	63	68.5%	1.02 [0.46, 2.28]
Kaneko 2003	7	63	2	38	12.9%	2.25 [0.44, 11.44]
Kawaski 1998	6	35	5	56	18.5%	2.11 [0.59, 7.52]
**Subtotal (95% CI)**		**160**		**157**	**100.0%**	**1.38 [0.74, 2.57]**
Total events	29		23			
Heterogeneity: Chi² = 1.31, df = 2 (P = 0.52); I² = 0%						
Test for overall effect: Z = 1.03 (P = 0.31)						
**1.3.5 Undifferentiated Histology**						
Ito 1996	41	62	27	63	22.3%	2.60 [1.26, 5.37]
Kaneko 2003	26	63	26	38	46.9%	0.32 [0.14, 0.76]
Kawaski 1998	20	35	38	56	30.8%	0.63 [0.26, 1.51]
**Subtotal (95% CI)**		**160**		**157**	**100.0%**	**0.93 [0.60, 1.44]**
Total events	87		91			
Heterogeneity: Chi² = 14.42, df = 2 (P = 0.0007); I² = 86%						
Test for overall effect: Z = 0.33 (P = 0.74)						
**1.3.6 Lymphatic Invasion**						
Ito 1996	40	62	42	63	60.4%	0.91 [0.43, 1.90]
Kaneko 2003	38	63	15	38	30.3%	2.33 [1.02, 5.31]
Kawaski 1998	31	35	26	56	9.3%	8.94 [2.79, 28.70]
**Subtotal (95% CI)**		**160**		**157**	**100.0%**	**2.09 [1.31, 3.34]**
Total events	109		83			
Heterogeneity: Chi² = 10.92, df = 2 (P = 0.004); I² = 82%						
Test for overall effect: Z = 3.08 (P = 0.002)						
**1.3.7 Vascular Invasion**						
Ito 1996	29	62	26	63	52.6%	1.25 [0.62, 2.54]
Kaneko 2003	26	63	10	38	28.1%	1.97 [0.82, 4.74]
Kawaski 1998	25	35	23	56	19.4%	3.59 [1.45, 8.88]
**Subtotal (95% CI)**		**160**		**157**	**100.0%**	**1.90 [1.20, 3.03]**
Total events	80		59			
Heterogeneity: Chi² = 3.24, df = 2 (P = 0.20); I² = 38%						
Test for overall effect: Z = 2.72 (P = 0.007)						

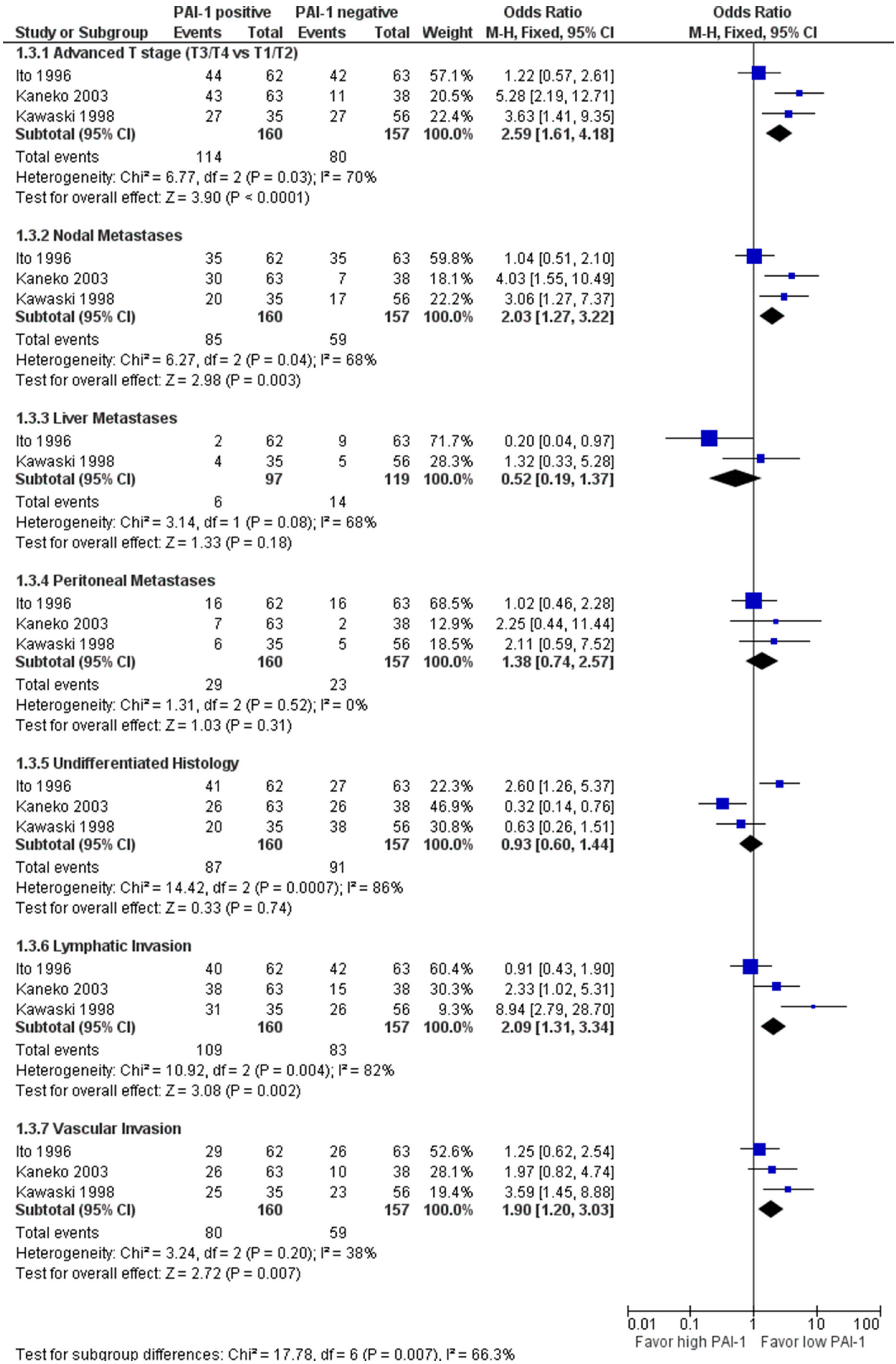

Test for subgroup differences: Chi² = 17.78, df = 6 (P = 0.007), I² = 66.3%

Supplementary Figure 4: Pooled estimate of odds ratio (OR) for PAI-1 expression and clinicopathological factors.

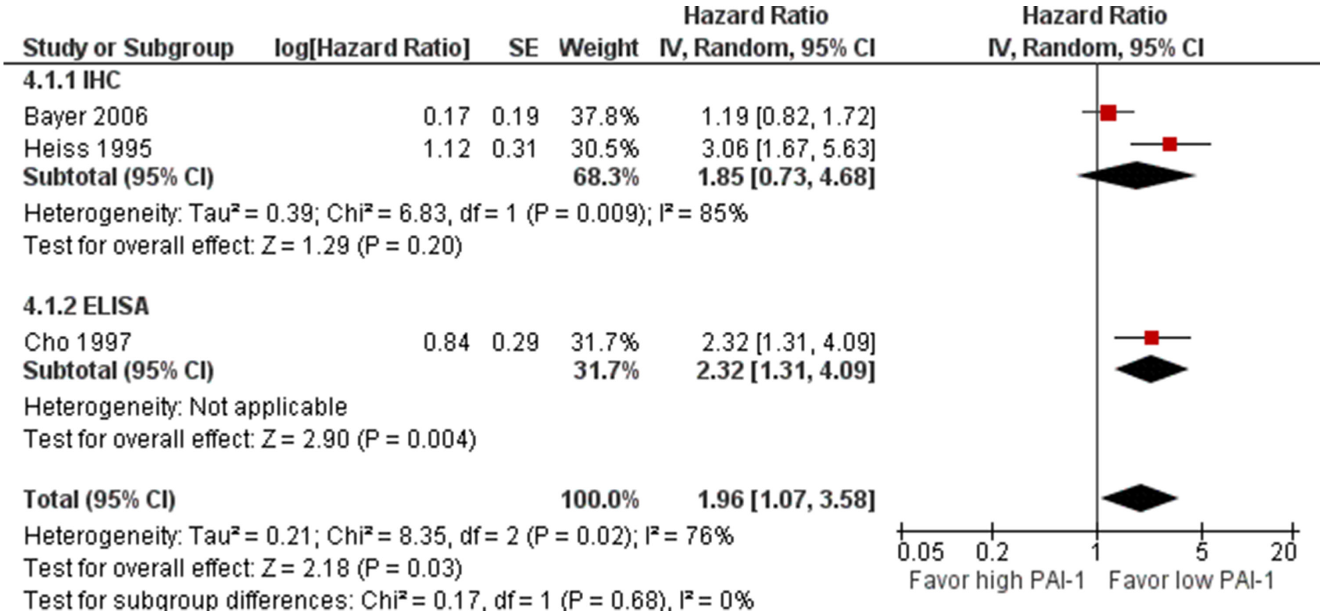

Supplementary Figure 5: Pooled estimate of hazard ratio (HR) for PAI-1 expression and recurrence free survival (RFS).

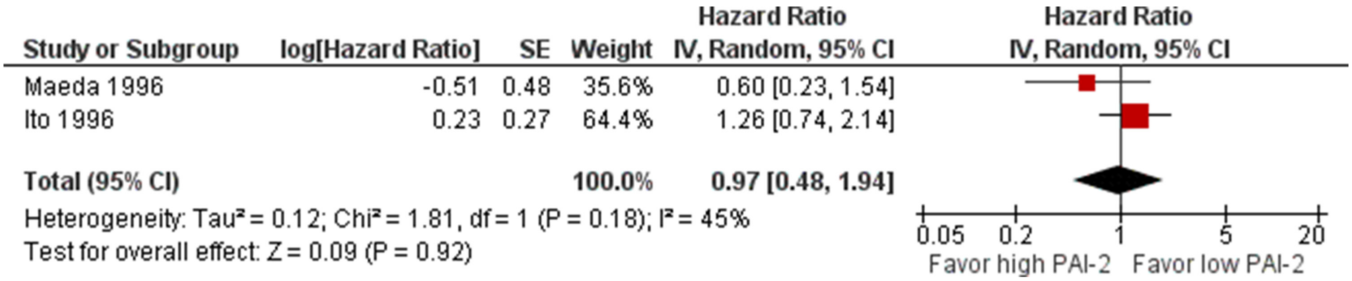

Supplementary Figure 6: Pooled estimate of hazard ratio (HR) for PAI-2 expression and overall survival (OS).

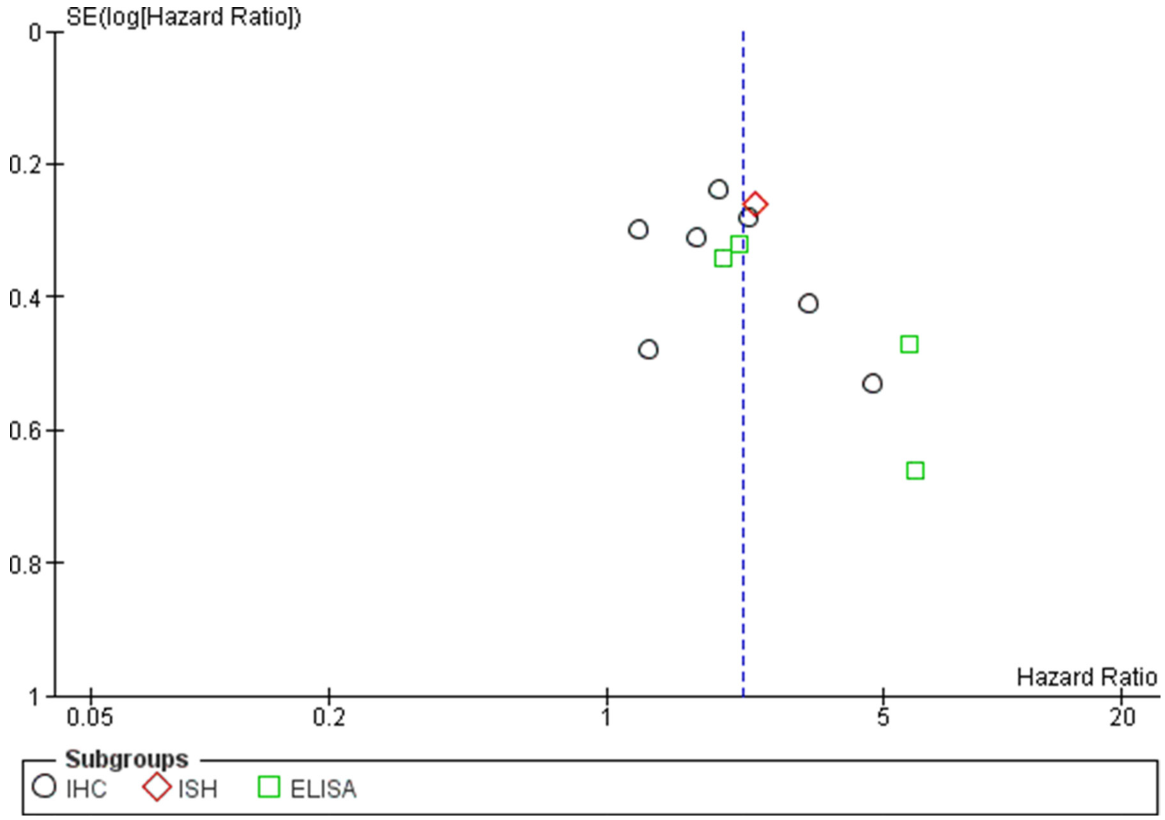

Supplementary Figure 7: Funnel plot of uPA studies.

Supplementary Table 1: Characteristics of included studies

	Study (year)	Pt No.	Primary location (TNM stage)	Analysis Method	uPA component	Expression Rate (%)
1	Allott (2012)[30]	39	Oesophageal (ND)	RT-PCR	PAI-1	50
2	Alpizar Alpizar (2012)[11]	95	Gastric (I–IV)	IHC	uPAR	90
3	Beyer (2006)[22]	104	Gastric (I–IV)	IHC	uPA	91
					uPAR	88
					PAI-1	90
4	Cho (1997)[27]	160	Gastric (I–IV)	ELISA	uPA	28
					PAI-1	33
5	Ganesh (1996)[29]	50	Gastric (I–IV)	ELISA	uPAR	42
					PAI-1	11
6	Heiss (1995)[42]	203	Gastric (I–IV)	IHC	uPA	75
					uPAR	69
					PAI-1	71
7	Ito (1996)[31]	125	Gastric (ND)	IHC	uPA	66
					PAI-1	50
					PAI-2	52
8	Kaneko (2003)[32]	101	Gastric (ND)	IHC	uPA	23
					uPAR	33
					PAI-1	37
9	Kawasaki (1998)[12]	91	Gastric (I – IV)	IHC	uPAR	33 (IHC)
				ISH		20 (ISH)
					PAI-1	39 (IHC)
						29 (ISH)
10	Lærum (2012)[7]	66	Oesophageal, GOJ, Gastric (I–IV)	IHC	uPAR	90*
11	Lee (2004)[33]	35	Gastric (I–IV)	RT-PCR	uPAR	63
12	Luebke (2006)[41]	105	Gastric (I–IV)	IHC	uPA	84
					PAI-1	93
13	Maeda (1996)[47]	120	Gastric (I – IV)	IHC	uPA	61
					PAI-2	63
14	Murata (1998)[35]	26	Gastric (IV)	IHC	uPA	42
15	Nekarda (1994)[48]	76	Gastric (I – IV)	ELISA	uPA	51
					PAI-1	41
16	Nekarda (1998)[28]	38	Oesophageal (I-IV)	ELISA	uPA	34
					PAI-1	32
17	Okusa (1999)[36]	71	Gastric (I–IV)	ELISA	uPA	50
18	Plebani (1997)[37]	20	Gastric (I–IV)	ELISA	uPA	65
					uPAR	65
					PAI-1	75
19	Sakakibara (2006)[38]	79	Gastric (I–IV)	RT-PCR	PAI-1	58
20	Taniguchi (1998)[39]	102	Gastric (ND)	IHC	uPAR	40
21	Yonemura (1997)[13]	155	Gastric (I–IV)	IHC	uPAR	14
22	Zhang (2006)[40]	105	Gastric (ND)	ISH	uPA	58
					uPAR	67

Pt No – number of patients; uPA – urokinase plasminogen activator; uPAR - urokinase plasminogen activator receptor; PAI-1 – plasminogen activator receptor 1; PAI-2 - plasminogen activator receptor 2; IHC – immunohistochemistry, RT-PCT - reverse transcription polymerase chain reaction; ISH – in-situ hybridisation; ELISA - enzyme-linked immunosorbent assay; GOJ – gastro-oesophageal junction.
*expression at invasive front
